# Supplementary material for: Structural insights into pathogenic mechanism of hypohidrotic ectodermal dysplasia caused by ectodysplasin A variants
Source: Nat Commun. 2023 Feb 11;14:767. doi: 10.1038/s41467-023-36367-6 (PMC9918506; doi:10.1038/s41467-023-36367-6)
Supplement: Supplementary file 1 — Supplementary Information [file 41467_2023_36367_MOESM1_ESM.pdf]

Supplementary Information

Structural insights into pathogenic mechanism of hypohidrotic ectodermal dysplasia caused by *Ectodysplasin A* variants

Kang Yu, Chenhui Huang, Futang Wan, Cailing Jiang, Juan Chen, Xiuping Li, Feng Wang, Jian Wu, Ming Lei and Yiqun Wu

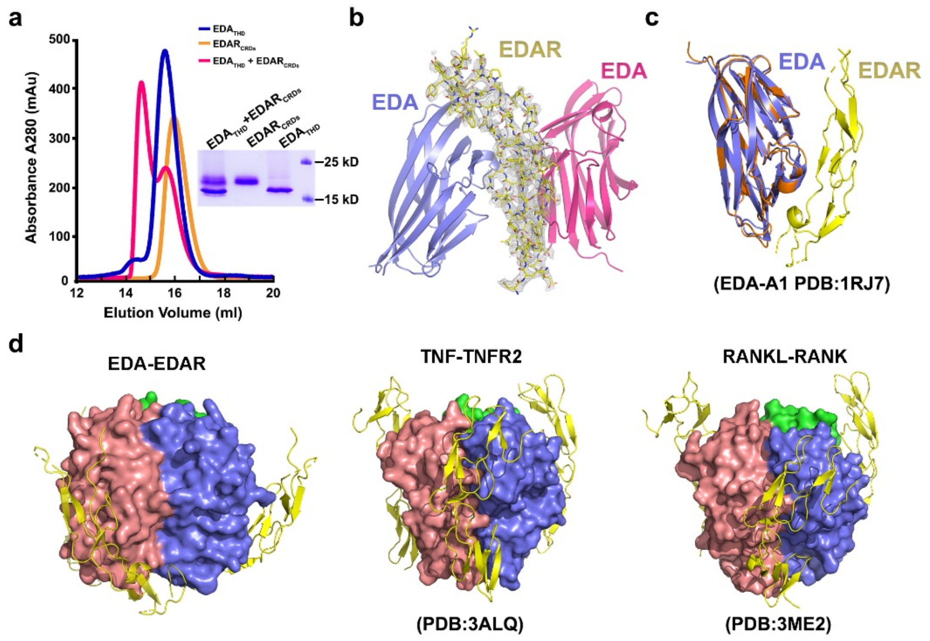

Supplementary Fig. 1 Crystal structure of the EDA·A1$_{THD}$-EDAR$_{CRDS}$ complex.

a Gel filtration profile of EDA·A1$_{THD}$-EDAR$_{CRDS}$ complex on a superdex 200 analytical column. The profile and the SDS-page gel show that purified EDA·A1$_{THD}$ and EDAR$_{CRDS}$ can form a stable complex. **b** The electron density map of EDAR$_{CRDS}$. The density map allows us to unambiguously trace the entire EDAR$_{CRDS}$ except for a few disordered loops. **c** Superposition of the apo monomeric EDA·A1$_{THD}$ (PDB: 1RJ7) onto the EDA·A1$_{THD}$-EDAR$_{CRDS}$ complex. The apo EDA·A1$_{THD}$ and EDA·A1$_{THD}$ in the complex are shown in cartoon and colored in orange and slate blue, respectively. **d** Comparison of EDA·A1$_{THD}$-EDAR$_{CRDS}$ with TNF$_{THD}$-TNFR2$_{CRDS}$ (PDB 3ALQ) and RANKL$_{THD}$-RANK$_{CRDS}$ (PDB 3ME2). Ligand protomers are shown in surface representation and colored in slate blue, green and orange for each chain, respectively. Receptor molecules are shown in cartoon representation and colored in yellow. EDAR$_{CRDS}$ binds to EDA·A1$_{THD}$ in a manner different from the other two published TNF family members.

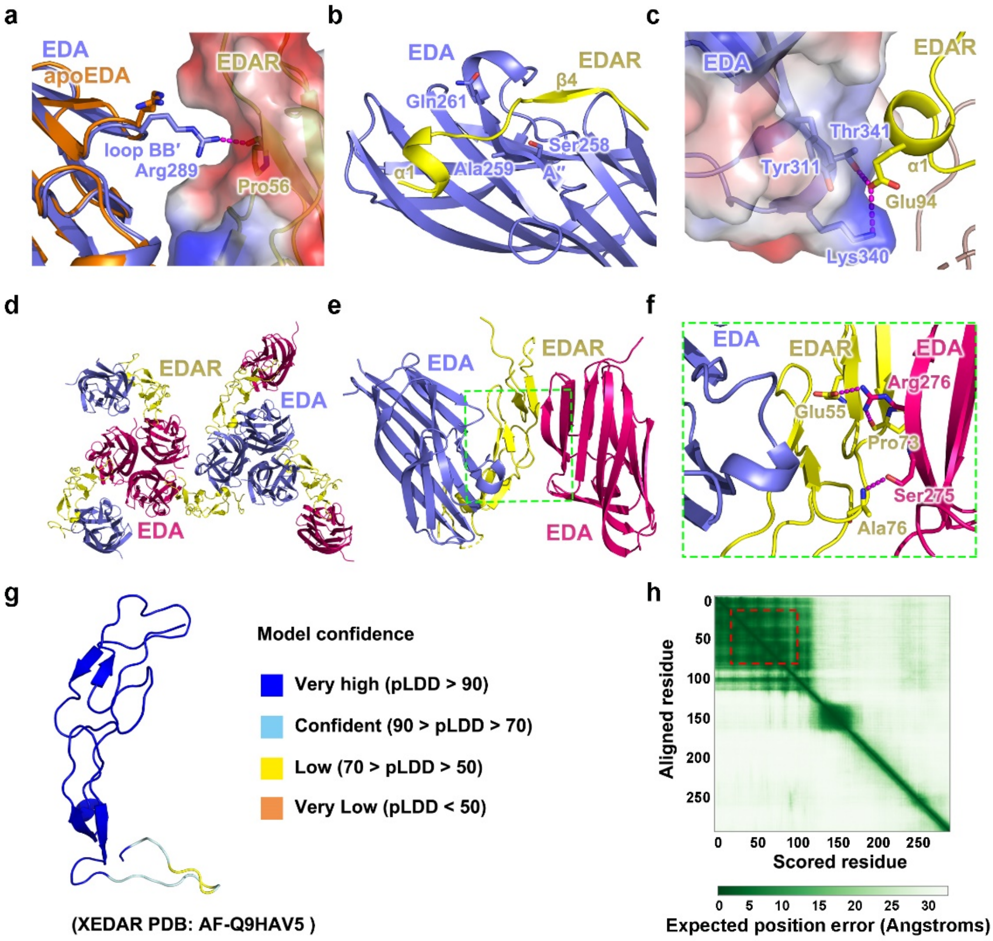

Supplementary Fig. 2 Comparison of apo- and receptor-bound EDA·A1$_{THD}$ structures.

a Superposition of the apo monomer EDA·A1$_{THD}$ and EDA·A1$_{THD}$-EDAR$_{CRDS}$ complex showing the side chain of Arg289$^{EDA·A1}$ points extending into a negatively charged depression within EDAR$_{CRD1}$ to contact with Pro56^{EDAR} upon binding to the receptor. **b** Interface area centered at Ala259$^{EDA·A1}$. The A'' stand of EDA·A1$_{THD}$ lies across the CRD2 loop L$_{45}$ (between strand β4 and helix α1) at Ala259. **c** Lys340 and Tyr341 of EDA·A1$_{THD}$ interact with Glu94^{EDAR} from two opposite directions, affecting the position and orientation of CRD2 helix α1. **d** The overall structure of the EDA·A1$_{THD}$-EDAR$_{CRDS}$ complex is shown in the Ribbon representation. **e** In an asymmetric unit, each EDAR$_{CRDS}$ exhibits interactions with two ligand protomers from two EDA·A1$_{THD}$ trimers. EDAR$_{CRDS}$ is colored in yellow, and ligand protomers in slate blue and warm pink, respectively. The interaction surface between EDA·A1$_{THD}$ and

$EDAR_{CRDS}$ is highlighted by a dashed rectangular box. **f** Close-up view of the interface between EDA·$A1_{THD}$ and $EDAR_{CRDS}$. Residues involved in the interaction are shown in stick models. **g** AlphaFold structure prediction of XEDAR. Left panel is the cartoon view of the predicted XEDAR (amino acid: 17-105) structure. Right panel is the model confidence. The XEDAR is color coded as the scheme shown. **h** Predicted aligned error of XEDAR. The color at position (x, y) indicates AlphaFold's expected position error at residue x, when the predicted and true structures are aligned on residue y. The red dash line box shows the amino acid of the XEDAR used for superposition.

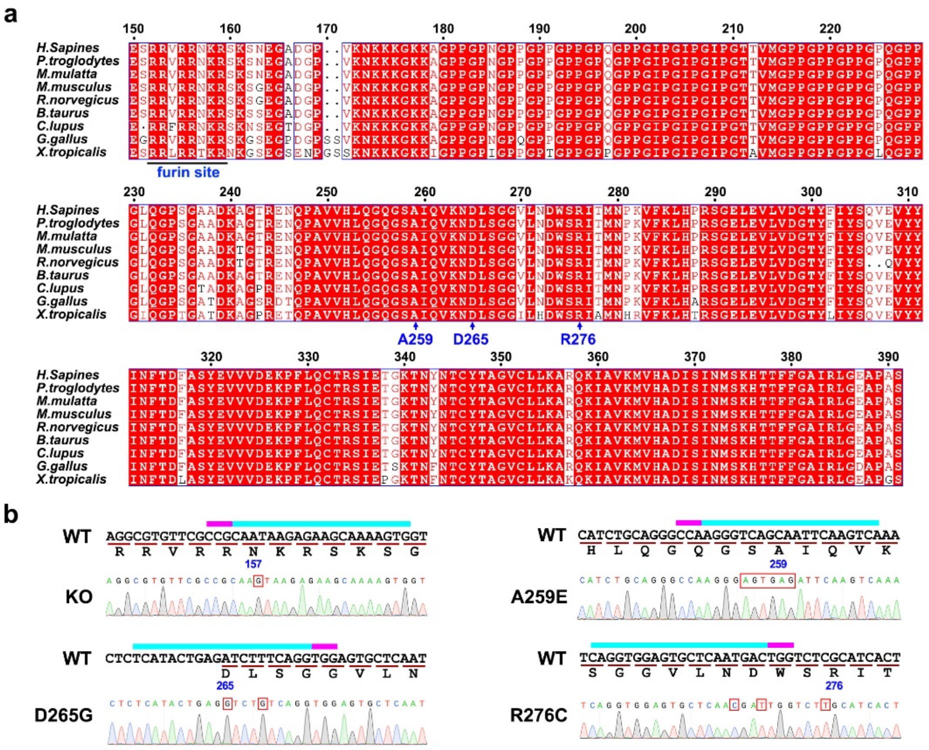

Supplementary Fig. 3 Targeted mutations of *Eda* in mice.

a Multiple sequence alignment of EDA furin sites and ectodomains from various species. Sequence positions are shown based on human EDA·A1. The furin recognition sequences and the desired mutation residues of A259, D265 and R276 are indicated. **b** Generation of knock-in mouse models via CRISPR-Cas9 and confirmation of mutations by tail biopsy of mouse offspring with PCR and DNA sequencing. The sgRNA-targeting site and the protospacer adjacent motif (PAM) are indicated above the sequence by cyan and pink bars, respectively. The desired mutation residues are numbered and the altered nucleotides are highlighted in red frames.

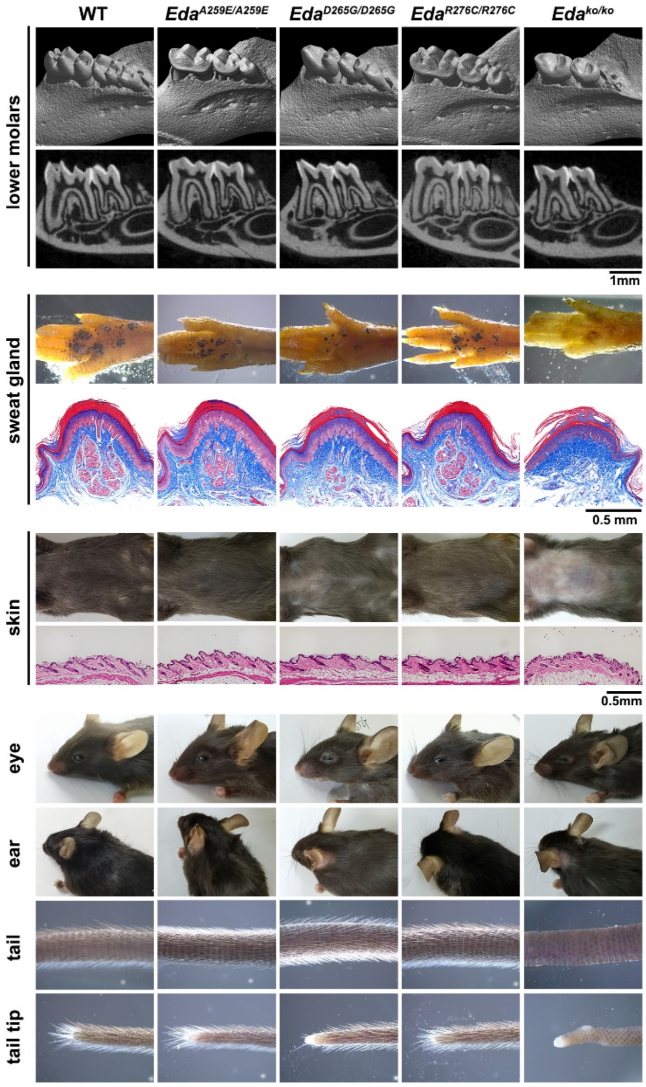

Supplementary Fig. 4 Deficiency of EDA causes similar ectodermal dysplasia in female mice with that of hemizygous male mice.

The *Eda* homozygous mutant female mice were analyzed for their ectodermal derivatives. The *Eda*$^{ko/ko}$ mice showed the most severe defects characterized by hairless tails and abdomen, kinked tail tips, a bald patch behind ears and abnormal eyelid development. The *Eda*$^{ko/ko}$ mice had only two mandibular molars, with lower M1 having only two rounded cusps and M2 exhibiting taurodontism. The *Eda*$^{ko/ko}$ mice showed no eccrine sweat glands in the footpads and displayed defects in sweating function revealed by the Starch-iodine test. The HED phenotypes of the *Eda*$^{D265G/D265G}$ mice were milder than that in the *Eda*$^{ko/ko}$ mice, showing tooth agenesis, defective

sweating abilities, scanty abdomen hair and ear hair, but with normal tail hair and tips. Although three mandibular molars were present in the *Eda*$^{D265G/D265G}$ mutant mice, lower M1 was small with only four of normal seven cusps formed, and M2 also exhibited taurodontism. The *Eda*$^{A259E/A259E}$ and *Eda*$^{R276C/R276C}$ mutant mice exhibited very mild tooth agenesis with molars displaying flattened, rounded cusps but no obvious abnormalities in tail tips, eyelid, tail hair, abdomen hair and hair behind ears.

Supplementary Table 1. Crystal data collection and refinement statistics.

	EDA_{THD}_$EDAR_{CRD}$
**Data collection**	
Wavelength (Å)	0.97853
Space group	*R*3
Cell dimensions	
a, b, c (Å)	87.2, 87.2, 156.4
α, β, γ (°)	90.0, 90.0, 120.0
Resolution (Å)	2.8
R_{merge} (%)	4.4 (69.6) *
$I / \sigma I$	26.1 (1.8) *
Completeness (%)	99.8 (99.8) *
Redundancy	3.5 (3.4) *
**Refinement**	
Resolution (Å)	34.71-2.80
No. of reflections	9,892
R_{work} / R_{free} (%)	24.8/28.2
No. of atoms	
EDA	2,243
EDAR	610
B-factors (Å^2)	
EDA	47.6
EDAR	73.4
R.m.s. deviations	
Bond lengths (Å)	0.011
Bond angles (°)	1.593
Ramachandran plot (%)	
Favored region	93.0
Allowed region	100.0
Outlier region	0.0

*Highest resolution shell is shown in parenthesis

Supplementary Table 2. Missense mutations in EDA·A1$_{THD}$.

EDA monomer interior (Type I)		
L253P	①	HED
V262F	②	HED
L266R	②	HED
L271P	①	HED
W274C	③	HED
W274G	③	HED
W274R	③	HED
M279R	③	HED
F284L	③	HED
G291R	②+③	HED
G291W	②	HED
L293R	②+③	HED
L293P	①	HED
Y301C	③	HED
S305I	③	HED
S305R	②+③	HED
V307G	①	HED
V309G	①	HED
I312M	④	NSTA
I312N	③	HED
Y320C	③	HED
V324E	③	HED
L330P	①	HED
C332F	②+③	HED
C332Y	②	HED
C346W	②+③	HED
C346Y	②	HED
T348A	③	HED
L354P	①	HED
I360N	③	HED
M364R	③	HED
M364T	④	NSTA
I371N	③	HED
M373I	④	HED
T378M	②+③	HED
T378P	①	HED
G381A	②	HED
G381R	②	HED
G381V	②	HED
I260S	④	NSTA

EDA trimer interaction surface (Type II)		
H252L	①	HED
H252N	①	HED
H252Q	①	HED
H252Y	①	HED
F302C	③	HED
F302S	③	HED
Y304C	③	HED
Y304H	③	HED
Y304N	③	HED
Q306H	②	HED
Q306P	① +②	HED
Q306R	②	HED
R334H	④	NSTA
A349T	②	HED
A349V	②	HED
G350C	③	HED
G350D	②+③	HED
V351F	②	HED
F379S	③	HED

EDA and EDAR interaction surface (Type III)		
A259E	③	NSTA
D265G	③	HED
R276C	③	HED
R289C	③	NSTA
R289H	③	NSTA
R289L	③	NSTA
R289P; S290C	①+③	HED
Y343C	③	HED
S370K	④	NSTA

EDA monomer exterior (Type IV)	
G255C	HED
G255D	HED
G257R	NSTA
G269V	HED
L296V	HED
D298H	HED
D298Y	HED
G299S	HED
G299A	HED
G299D	HED
G299R	HED
D316N	HED
D316E	HED
D316G	NSTA
S319R	HED
S319I	NSTA
V323G	HED
Q331H	NSTA
T338M	NSTA
A356D	HED
A356V	HED
R357P	HED
R357W	HED
K363E	HED
V365A	NSTA
N372D	HED
N372K	HED
S374R	HED
T376M	HED
R384S	HED
P389L	NSTA

Classification of *EDA* mutations

Type I: Mutations in the interior of EDA·A1$_{THD}$ which cause protein destabilization.

Type II: Mutations at the EDA·A1 trimeric interface which affect the trimer formation.

Type III: Mutations at the ligand-receptor interface which interfere with the interaction with EDAR.

Type IV: Mutations located at the exterior of EDA·A1$_{THD}$ away from the ligand-receptor interface, which lack hints on their biochemical consequences.

Severity of diseases

HED: Hypohidrotic ectodermal dysplasia, congenital syndrome characterized by sparse hair, oligodontia, and reduced sweating.

NSTA: non-syndromic tooth agenesis that only affects the dentition.

Predicated deleterious effects

① Mutations that change the local geometry.

② Mutations that introduce a large sidechain to cause steric hindrance.

③ Mutations that disrupt hydrophobic or hydrophilic interactions.

④ Mutations that have mild changes in physico-chemical properties.

Supplementary Table 3. List of primers used for gene cloning, site-directed mutagenesis, and construction and genotyping of mutant mice.

**Gene cloning**	
EDA-opt-233-391-F	5'-CGCGGATCCGGTCCTAGTGGCGCCGCTG-3'
EDA-opt-233-391-R	5'-CGCCTCGAGACTGGCCGGGGCTTCACCC-3'
EDA-233-391-F	5'-CGCGGATCCGGACCTTCTGGTGCTGCTGATAAAG-3'
EDA-233-391-R	5'-CGCCTCGAGGGATGCAGGGGCTTCACCCAG-3'
EDAR-30-150-F	5'-CGCGGATCCAACTGCGGTGAGAACGAG-3'
EDAR-30-150-R	5'-CGCCTCGAGTCCCACACATTCCTTGGTG-3'
EDAR-1-448-F	5'-CGCGGATCCATGGCCCATGTGGGGGA-3'
EDAR-1-448-R	5'-CGCTCTAGAGGATGCAGCATGTGGCTG-3'
**Site-directed mutagenesis**	
EDA-A259E-opt-F	5'-AGGGTCAGGGTAGTGAAATTCAGGTTAAAAATGATC-3'
EDA-A259E-opt-R	5'-GATCATTTTTAACCTGAATTTCACTACCCTGACCCT-3'
EDA-D265G-opt-F	5'-AGTGCCATTCAGGTTAAAAATGGTCTGAGCGGCGG-3'
EDA-D265G-opt-R	5'-CCGCCGCTCAGACCATTTTTAACCTGAATGGCACT-3'
EDA-R276C-opt-F	5'-GTGCTGAATGATTGGAGTTGCATTACCATGAATCCG-3'
EDA-R276C-opt-R	5'-CGGATTCATGGTAATGCAACTCCAATCATTCAGCAC-3'
EDA-A259E-F	5'-GGCCAAGGGTCAGAAATTCAAGTCAAGAATGATC-3'
EDA-A259E-R	5'-GATCATTCTTGACTTGAATTTCTGACCCTTGGCC-3'
EDA-D265G-F	5'-GCAATTCAAGTCAAGAATGGTCTTTCAGGTGGAGTGC-3'
EDA-D265G-R	5'-GCACTCCACCTGAAAGACCATTCTTGACTTGAATTGC-3'
EDA-R276C-F	5'-GCTCAATGACTGGTCTTGCATCACTATGAACCCC-3'
EDA-R276C-R	5'-GGGGTTCATAGTGATGCAAGACCAGTCATTGAGC-3'
**Construction and genotyping of mutant mice**	
gRNA-KO	5'-CACTTTTGCTTCTCTTATTG-3'
gRNA-A259E	5'-TGACTTGAATTGCTGACCCT-3'
Donor-A259E	5'-GAAACTCCTAGGAGGGAGACATTCTACTTTGATTCTTACCATTTT TGACTTGAATCTCACTCCCTTGGCCCTGCAGATGCACCACAGCTGG CTGAGAAGATGAGAAGAAGAGAGTC-3'
gRNA-D265G	5'-TCATACTGAGATCTTTCAGG-3'
Donor-D265G	5'-CTGTTGCCCGAATTATTTTGACATGTACTGAGTGACTGCCCTTCT CTCATACTGAGGTCTGTCAGGTGGAGTGCTCAATGACTGGTCTCGC ATCACTATGAACCCTAAGGTGTTTAAAC-3'
gRNA-R276C	5'-CAGGTGGAGTGCTCAATGAC-3'
Donor-R276C	5'-CTCCAGCTCCCCGCTGCGGGGATGTAGTTTAAACACCTTAGGGTT CATAGTGATGCAAGACCAATCGTTGAGCACTCCACCTGAAAGATCT CAGTATGAGAGAAGGGCAGTCACTCAG-3'
mEDA-KO-F	5'-GTGGACAAATGTAACCTACGGGAC-3'
mEDA-KO-R	5'-GTCCTTGTTGAGAGTAACCTTG-3'
mEDA-A259E-F	5'-AGAGTCCTATAGTTCCCGCTTTAC-3'
mEDA-A259E-R	5'-ATGTTTGGTTAGCATTCACGAGGC-3'
mEDA-D265G/R276C-F	5'-GCCACCTCAGATTCCAACTAGAAG-3'
mEDA-D265G/R276C-R	5'-AACTCCCTCCCAGTGTGAAATCGG-3'
