# Supplementary material for: Structural insights into pathogenic mechanism of hypohidrotic ectodermal dysplasia caused by ectodysplasin A variants
Source: Nat Commun. 2023 Feb 11;14:767. doi: 10.1038/s41467-023-36367-6 (PMC9918506; doi:10.1038/s41467-023-36367-6)
Supplement: Supplementary file 3 — Reporting Summary [file 41467_2023_36367_MOESM3_ESM.pdf]

nature portfolio

Corresponding author(s): Yiqun Wu, Ming Lei, Jian Wu

Last updated by author(s): Jan 5, 2023

Reporting Summary

Nature Portfolio wishes to improve the reproducibility of the work that we publish. This form provides structure for consistency and transparency in reporting. For further information on Nature Portfolio policies, see our Editorial Policies and the Editorial Policy Checklist.

Statistics

For all statistical analyses, confirm that the following items are present in the figure legend, table legend, main text, or Methods section.

n/a	Confirmed	
☐	☒	The exact sample size (*n*) for each experimental group/condition, given as a discrete number and unit of measurement
☐	☒	A statement on whether measurements were taken from distinct samples or whether the same sample was measured repeatedly
☐	☒	The statistical test(s) used AND whether they are one- or two-sided<br>*Only common tests should be described solely by name; describe more complex techniques in the Methods section.*
☒	☐	A description of all covariates tested
☒	☐	A description of any assumptions or corrections, such as tests of normality and adjustment for multiple comparisons
☐	☒	A full description of the statistical parameters including central tendency (e.g. means) or other basic estimates (e.g. regression coefficient) AND variation (e.g. standard deviation) or associated estimates of uncertainty (e.g. confidence intervals)
☐	☒	For null hypothesis testing, the test statistic (e.g. *F*, *t*, *r*) with confidence intervals, effect sizes, degrees of freedom and *P* value noted<br>*Give P values as exact values whenever suitable.*
☒	☐	For Bayesian analysis, information on the choice of priors and Markov chain Monte Carlo settings
☒	☐	For hierarchical and complex designs, identification of the appropriate level for tests and full reporting of outcomes
☒	☐	Estimates of effect sizes (e.g. Cohen's *d*, Pearson's *r*), indicating how they were calculated

Our web collection on statistics for biologists contains articles on many of the points above.

Software and code

Policy information about availability of computer code

Data collection	no software was used.
Data analysis	Coot version 0.89 (Emsley and Cowtan, 2004)<br>Phenix version 1.19.2 (http://www.phenix-online.org/)<br>PyMOL version 1.86 (Schrödinger)<br>Imaris version 9.2 (Oxford Instruments)<br>GraphPad Prism v8.0 (GraphPad)<br>Biacore 8K Evaluation Software version 1.0

For manuscripts utilizing custom algorithms or software that are central to the research but not yet described in published literature, software must be made available to editors and reviewers. We strongly encourage code deposition in a community repository (e.g. GitHub). See the Nature Portfolio guidelines for submitting code & software for further information.

Data

Policy information about availability of data

All manuscripts must include a data availability statement. This statement should provide the following information, where applicable:

- Accession codes, unique identifiers, or web links for publicly available datasets
- A description of any restrictions on data availability
- For clinical datasets or third party data, please ensure that the statement adheres to our policy

The crystal structure of the EDA-EDAR complex was deposited in PDB with accession code 7X9G. All data needed to evaluate the conclusions in the paper are present in the paper and/or the Supplementary Materials. The database used in this study includes PDB 1RJ7, 1RJ8, 3ALQ, 3ME2 and AF-Q9HAV5. Source data are provided with this paper.

Human research participants

Policy information about studies involving human research participants and Sex and Gender in Research.

Reporting on sex and gender	Not Applicable.
Population characteristics	Not Applicable.
Recruitment	Not Applicable.
Ethics oversight	Not Applicable.

Note that full information on the approval of the study protocol must also be provided in the manuscript.

Field-specific reporting

Please select the one below that is the best fit for your research. If you are not sure, read the appropriate sections before making your selection.

☒ Life sciences ☐ Behavioural & social sciences ☐ Ecological, evolutionary & environmental sciences

For a reference copy of the document with all sections, see nature.com/documents/nr-reporting-summary-flat.pdf

Life sciences study design

All studies must disclose on these points even when the disclosure is negative.

Sample size	For animal experiments, no statistical method was used to predetermine sample size, and at least n=3 mice of each genotype were analyzed. This was shown to be sufficient in previous studies (e.g., Xu, M. et al Nat Commun 2017, Fernandez-Guerrero, M. et al Proc Natl Acad Sci U S A 2020) to discern statistically significant differences. In molecular biology experiments, n>=3 was chosen to generate p-values to determine if results are significant.
Data exclusions	No data were excluded.
Replication	The in vitro data were reproduced in technical and biological duplicates and experiments were performed at least three times. The exact number of mice used in each group in an experiment is mentioned in Statistics and Reproducibility section and corresponding figure legends. All attempts at replication were successful.
Randomization	For mouse analysis, we are comparing phenotypes between WT and Eda mutant pups. Mice and sections used for imaging were selected randomly. For cell culture-based assays, a common pool of cells were randomized (i.e. aliquoted) equally among treatment groups.
Blinding	Experimental set-up and data analysis were performed by the same individuals. All mouse samples were labeled with numerical identifiers (i.e. animal number) rather than experimental groups, which were revealed until data interpretation.

Reporting for specific materials, systems and methods

We require information from authors about some types of materials, experimental systems and methods used in many studies. Here, indicate whether each material, system or method listed is relevant to your study. If you are not sure if a list item applies to your research, read the appropriate section before selecting a response.

Materials & experimental systems

n/a	Involved in the study	
☐	☒	Antibodies
☐	☒	Eukaryotic cell lines
☒	☐	Palaeontology and archaeology
☐	☒	Animals and other organisms
☒	☐	Clinical data
☒	☐	Dual use research of concern

Methods

n/a	Involved in the study	
☒	☐	ChIP-seq
☒	☐	Flow cytometry
☒	☐	MRI-based neuroimaging

Antibodies

Antibodies used	The following antibodies were used in this study: secondary antibodies for IF and western blotting: Alexa Fluor 594 Goat anti-Rabbit IgG (Invitrogen, A-11012, 1:500, RRID: AB_2534079), Alexa Fluor 488 Goat anti-Mouse IgG (Invitrogen, A-11001, 1:500, RRID: AB_2534069), HRP-conjugated Goat anti-Mouse IgG (Proteintech, SA00001-1, 1:4000, RRID: AB_2722565), and HRP-conjugated Goat anti-Rabbit IgG (Proteintech, SA00001-2, 1:4000, RRID: AB_2722564). Rabbit antibodies against FLAG (Proteintech, 20543-1, 1:2000, RRID: AB_11232216). Mouse antibodies against MBP (Proteintech, 66003-1, 1:2000, RRID: AB_11183040).
Validation	Activities of Alexa Fluor 594 Goat anti-Rabbit IgG (Invitrogen, A-11012) is validated via https://www.thermofisher.cn/cn/zh/antibody/product/Goat-anti-Rabbit-IgG-H-L-Cross-Adsorbed-Secondary-Antibody-Polyclonal/A-11012;<br>Activities of Alexa Fluor 488 Goat anti-Mouse IgG (Invitrogen, A-11001) is validated via https://www.thermofisher.cn/cn/zh/antibody/product/Goat-anti-Mouse-IgG-H-L-Cross-Adsorbed-Secondary-Antibody-Polyclonal/A-11001;<br>Activities of HRP-conjugated Goat anti-Mouse IgG (Proteintech, SA00001-1) is validated via https://www.ptgcn.com/products/HRP-conjugated-Affinipure-Goat-Anti-Mouse-IgG-H-L-secondary-antibody.htm<br>Activities of HRP-conjugated Goat anti-Rabbit IgG (Proteintech, SA00001-2) is validated via https://www.ptglab.co.jp/products/HRP-conjugated-Affinipure-Goat-Anti-Rabbit-IgG-H-L-secondary-antibody.htm<br>Activities of rabbit anti-Flag antibody (Proteintech, 20543-1) is validated for Flag-tag fusion protein (Applications: IF, WB, ELISA) via https://www.ptgcn.com/products/Flag-Tag-Antibody-20543-1-AP.htm<br>Activities of mouse anti-MBP antibody (Proteintech, 66003-1) is validated for MBP-tag fusion protein (Applications: IF, WB, ELISA) via https://www.ptgcn.com/products/MBP-Tag-Antibody-66003-1-Ig.htm

Eukaryotic cell lines

Policy information about cell lines and Sex and Gender in Research

Cell line source(s)	HEK293T and HaCaT cells were purchased from the Type Culture Collection of the Chinese Academy of Sciences, Shanghai. China.
Authentication	Cells were authenticated by the cell bank of type culture collection of Chinese Academy of Sciences using STR DNA profiling.
Mycoplasma contamination	No mycoplasma contamination was detected in HEK293T and HaCaT cells used in this study.
Commonly misidentified lines (See ICLAC register)	No commonly misidentified cell lines were used.

Animals and other research organisms

Policy information about studies involving animals; ARRIVE guidelines recommended for reporting animal research, and Sex and Gender in Research

Laboratory animals	Male and female C57BL/6 mice between 6-12 and ICR(CD-1) mice between 8-12 weeks were used in this study. Fertilized eggs collected from C57BL/6 mice were used for generation of Eda mutant mice by CRISPR/Cas-mediated genome engineering. Mutant mice were congenic with the C57BL/6 background. Tissues from mice between 6-8 weeks were used in this study.
Wild animals	No wild animals were used in the study.
Reporting on sex	For animal experiments, both male and female mice were used in this study.
Field-collected samples	No field collected samples were used in the study.
Ethics oversight	All procedures involving mice and experimental protocols were approved by the Institutional Animal Care and Research Advisory Committee of the Shanghai Ninth People's Hospital, School of Medicine, Shanghai Jiao Tong University (approval # SH9H-2019-A130-1).

Note that full information on the approval of the study protocol must also be provided in the manuscript.
